# Disentangling Stigma from Functional Neurological Disorders: Conference Report and Roadmap for the Future

**DOI:** 10.3389/fneur.2017.00106

**Published:** 2017-03-29

**Authors:** Karen S. Rommelfanger, Stewart A. Factor, Suzette LaRoche, Phyllis Rosen, Raymond Young, Mark H. Rapaport

**Affiliations:** ^1^Department of Psychiatry and Behavioral Sciences, Emory University, Atlanta, GA, USA; ^2^Department of Neurology, Emory University, Atlanta, GA, USA; ^3^Center for Ethics Neuroethics Program, Emory University, Atlanta, GA, USA; ^4^Mission Health, Epilepsy Center, Asheville, NC, USA

**Keywords:** psychogenic, conversion, functional neurological disorders, medically unexplained illness, stigma, education, neuroethics, multidisciplinary care

## Abstract

A multidisciplinary expert review of key issues and future directions from the conference “Controversial labels and clinical uncertainties: psychogenic disorders, conversion disorder, and functional symptoms.”

On October 9 and 10, 2015, a conference entitled “*Controversial labels and clinical uncertainties: psychogenic disorders, conversion disorder, and functional symptoms*” was held at the Center for Ethics, Emory University, Atlanta, GA, USA. This conference brought together a select group of 30 distinguished thought leaders and practitioners, including ethicists, researchers, clinicians, humanities scholars, and advocates to discuss the unique challenges and controversies related to the diagnosis, treatment, and stigma for patients with what is currently recognized as functional (“psychogenic”) neurological disorders. Our group of experts explored the conflicts and ethical tensions within health care that must be addressed in order to advance care for these disorders. What follows is a reflection on the conversations between conference attendees outlining key challenges and value conflicts in the diagnosis and treatment of patients with functional disorders. With this report, we aim to provide a roadmap for reducing stigma and improving care for functional neurological disorders (FND). A path forward would involve (1) setting a multifactorial research agenda that equally prioritized access to effective psychotherapy as well as identification of novel biomarkers; (2) empowering patients with FND to be heard and to drive changes in care; and (3) reducing isolation for clinicians by providing formal training and setting up multidisciplinary care teams and support networks.

## Introduction

Functional neurological disorders (FND) are conceptualized as a manifestation of neurological symptoms that arise from a psychiatric origin. FND represents a confounding situation where an otherwise invisible illness becomes visible. As Jakes story illustrates (Box 1), FND lacks ownership; an orphan to a disciplinary home in medicine, falling in the netherworld of thew neurology-psychiatry abyss. The high prevalence, poor prognosis, lack of available treatments, and the fact that patients often have disbelief in their diagnosis has lead to a “A crisis for neurology” (1). However, *any* clinician who find themselves in the position of caring for such patients are faced with a dearth of resources including a lack of formal training, guidelines, standards of care, referral options, and emotional support. Because of this need to address, the overwhelming challenges of FND, on October 9 and 10, 2015, “*Controversial labels and clinical uncertainties: psychogenic disorders, conversion disorder, and functional symptoms*” was held at the Center for Ethics, Emory University, Atlanta, GA, USA. This conference brought together a selected group of 30 distinguished thought leaders in the field including ethicists, researchers, clinicians, humanities scholars, and advocates to discuss the unique challenges and controversies related to the diagnosis, treatment, and stigma for patients with functional (“psychogenic”) disorders. Conference attendees aimed to outline key challenges in the diagnosis and treatment of patients with functional disorders. We asked: *What value conflicts exist across healthcare professionals and patients that create hurdles for care? How can biased attitudes affect diagnosis and treatment? What are the research priorities needed for developing a standard of care? What controversies require further exploration in a larger forum?* Within the framework of these four questions, we utilized category prompts (Box S1 in Supplementary Material) to focus our discussions on diagnosis, treatment, physician training, and infrastructure challenges. In this report, we, the conference organizers, aim to describe, summarize, synthesize many varied voices that were represented at the conference. What follows is a reflection, from the authors, on these conversations and a roadmap guided by the conversations with conference attendees, for reducing stigma and advancing care for FND.

Box 1 **Patient Narrative.**

Jake suddenly developed a constellation of symptoms including stumbling gait, jerking movements, and impaired speech. Alarmed and concerned, his family rushed him to the emergency department (ED). The ED physician told him awkwardly that they could find no abnormalities on his evaluation, that they could not explain his illness, and recommended a neurologist. The neurologist indicated to him that his symptoms were likely psychological and that in fact, this was good news because it was not serious. He was then instructed to see a psychiatrist. Jake reluctantly went to the psychiatrist who said, “You don’t need to see me; you have no clear psychiatric illness yet very clear physical manifestations.” Jake left feeling embarrassed and angry, believing “the docs think this is all in my head and that I’m making this up.” Jake continued to see many providers looking for an answer. They appeared to dread seeing him and wondered if he was malingering or feigning illness. Jake and his family were left feeling frustrated and helpless. His symptoms remained unresolved, and he has now amassed tremendous health-care expenditures.

### About FND

There is no known biological etiology for FND, and the lack of mechanistic understanding is reflected in the terminologies used by neurologists and psychiatrists and lack of clear disciplinary home for FND. The term “psychogenic,” most preferred by neurologists according to a 2009 survey (2), describes disorders manifesting with physical conditions that cannot be attributed to underlying organic pathology (3, 4). A direct etymylogical interpretation suggests a genesis within the psyche or soul revealing the admonishing undertones to those who have come to be afflicted with a sick soul. While some neurologists have advocated for a change from “psychogenic” to “functional” (5), there are others who argue that “functional” introduces more ambiguity with historical roots intertwined with terms like “hysteria” (6). Psychiatrists may refer to these disorders as conversion disorder, but in our conference, psychiatrists were quick to remind us that a “psychogenic” diagnosis is not synonymous with conversion because they do not necessarily meet Diagnostic and Statistical Manual of Mental Disorders (DSM) criteria. For the purpose of this paper, we choose to use the term “functional” in deference to the language of choice of patient advocacy organizations Functional Neurological Disorders Hope (FNDHope) and ProjectFND.

Functional movement disorders (FMDs) are among the more common FND (3), affecting up to 25% of patients who visit specialized movement disorder clinics (7). Epidemiological studies suggest that some FMDs, such as functional tremor, have a higher incidence than “organic” neurological disorders such as multiple sclerosis or amyotrophic lateral sclerosis [for review, see Nowak and Fink (3)] and experience worse quality of life and severity of disability scores compared to “organic” movement disorders including Parkinson’s disease (8). Long-term follow-up studies reveal a poor prognosis with 50–90% of FMD patients experiencing ongoing symptoms (9–11), many of whom become worse, especially when treatment begins later than 6–12 months from symptom onset (12, 13). Non-epileptic seizures, are not associated with epileptic discharges, yet manifest paroxysmal, involuntary alterations in motor, sensory and/or behavioral function that resemble epileptic seizures (14). Functional (psychogenic) non-epileptic seizures (PNES), like FMDs, are presumed to be caused by psychological distress and have been reported to affect up to 30% of patients who are referred to epilepsy centers (15, 16). Quality of life scores are often worse than patients with refractory epilepsy (17). Patients with FND often experience multiple referrals, frequent office visits, as well as expensive, sophisticated, and invasive tests (8). It is important to note that FND have many other motor and sensory manifestations such as weakness (18) and paralysis (3) to name a few. The estimated annual US healthcare costs from FMD alone is at least $20 billion (19, 20).

### False Distinctions: The Not-So-Bright Line between Neurology and Psychiatry

The crisis of FND is a crisis of ideology, a direct product of a spuriously perceived division between neurology and psychiatry. Neurologists take care of the body and psychiatrists tend to the mind. Such a separation has had a negative impact on treatment, by creating tangible health-care infrastructure challenges and gaps in care, and neglect from funding agencies, making FND orphan to a medical treatment home. The disciplinary divide holds explanatory power to patients and clinicians alike. A different kind of moral judgment is cast depending upon whether one conceptualizes FND as etiologically a neurological or mental disorder. If the patient has a diseased mind, the patient is more blameworthy for the illness; if the patient has a diseased body, then the patient is less responsible (21). In fact, *the DSM* (22) distinguishes functional disorders (conversion disorder) from intentional maladies such as malingering or factitious disorder, however, neurologists do not universally accept this distinction with the conviction that a patients’ conscious intention to manifest in functional symptoms is not possible to determine or differentiate (4, 12). A rich discussion on policing this divide continues to brew significant discontent in the medical community today (23–25). There is so much allegiance to this division that any attempt to blur the line between neurology and psychiatry is cause for unrest, a veritable affront to the natural moral and social order.

### Stigma and FND

In the case scenario (Box S1 in Supplementary Material), we see that Jake is left feeling confused and frustrated. Despite the neurologist’s view that the diagnosis is “good news,” he is told he should see a mental health-care provider. Why the mismatch? For those with FND, there is a stigma ascribed to having mental illness as the cause of their symptoms. Stigma is a cognitive framing or worldview that leads to stereotyping, prejudice, and unjust discriminatory acts (26). With stigma, negative evaluations lead to negative emotions. Ultimately, these negative evaluations lead to punitive measures such as removing rightful opportunities (in the case of FND, appropriate health care), avoidance of stigmatized parties, and overall deleterious treatment of those who are the subject of the stigma (26, 27).

Stigma, represented by three forms: public stigma, self-stigma, and avoidance for the label itself (26), is eminently present in those with FND. Public stigma is represented by the avoidance and withdrawal of many clinicians from patients with FND. Patients with FND are seen as a risk, requiring an inordinate amount of time (at least more than physicians may be typically allotted for routine patients) and introduce personal discomfort in physicians who feel they have lack of expertise and resources to properly care for these patients. There is also fear of litigation. Self-stigma, wherein the subject of the stigma internalizes the prejudice and discrimination, is also a significant hurdle for FND. It is clear that the reluctance to accept a diagnosis of “psychogenic,” “conversion,” or any derivative thereof, as well as avoidance of mental health care, is active efforts to distance oneself from a label that patients fear is socially unacceptable. There is no label for FND that will eliminate stigma. Eliminating stigma requires more than revising our terminology. Stigma is about the pernicious associated meaning of undesirable difference and otherness that leads to prejudice, discrimination, and social exclusion. The label is only a signifier, and the meaning will follow regardless of a new name.

### Can a Biological Explanation Eliminate Stigma from FND?

Suggesting that mental illness is a biological disorder has, according to some, at least partially mitigated stigma (21). Rebranding psychiatry by conceptualizing “disorders of the mind as disorders of the brain” has been advocated (28). The hope with such a strategy is that an authoritative representation of a biological disorder will somehow legitimize (or perhaps materialize) the patient’s symptoms and suffering (29). However, a number of studies have suggested that even with increased knowledge about mental illnesses and their status as “brain disorders,” stigma has been relatively unchanged over the past two decades (30). Attributing mental illness to a biological origin brings its own brand of stigma (i.e., assumptions that people who are mentally ill are biological weaker, cannot recover, and are perhaps even dangerous) (26).

Choudhury et al. (31) warned against the oversimplification of the mental as merely biological phenomena, “the reduction of psychiatry to neurobiology tends to neglect the phenomenological insights, biographical accounts of the person and the *meaning*—that is, the social, cultural, moral, or spiritual significances—of mental illness or interventions” (31). FND requires a more holistic psychosocial–biological model rather than reducing people to, as Dumit says, merely “objects of science and medicine” (32). This is not to say that as more biological correlates of FND are discovered they should be ignored. Recent neuroimaging data suggests that FMD patients suffer from sensory deficits and impaired perceived “voluntariness” (3, 33), while patients with PNES have evidence of altered functional and structural connectivity (34). While this work is in its infancy, these findings can help patients and clinicians have fruitful conversations that disrupt assumptions about the nature of FND. As comforting as employing biological explanations for these disorders may be, these correlates are not the panacea to stigma. Such a strategy can implicitly endorse a classification system that would, in turn, oversimplify these disorders and foreclose a more holistic view while preventing a multidisciplinary treatment approach.

## Approaches to Disrupt Boundaries, Reduce Stigma, and Advance Care

### Encourage Multidisciplinary Research

To transform care, FND must be appreciated in its full complexity: psychosocial–biological. Working toward a multidisciplinary research agenda is a critical step toward advancing diagnosis, treatment, and long-term care for patients with FND. Interventions, thus far, have been wide-ranging including psychodynamic therapy, hypnosis, cognitive behavioral therapy, biofeedback, physical therapy, transcranial magnetic stimulation, and medications including antidepressants (3, 35); however, there is a striking paucity of randomized controlled trials (36–40). Resources should be aimed at making psychological therapy options more accessible while providing opportunities to gain biological insights. Identifying biomarkers utilizing clinical neurophysiology (EEG/EMG) and neuroimaging [fMRI and DATscans (41)] must be given equal priority to research on psychotherapeutic treatment options (i.e., cognitive behavioral therapy, dialectical behavior therapy, and supportive therapy).

It is clear that we are far from identifying the most effective treatment approach given the heterogeneity of the FND. But it is agreed upon that treatment must be individualized. Goals must also be constructed with patients and their families to provide insight into the treatment plan and feedback of successful communication strategies between clinicians and patients must be maintained throughout the process (42). These goals may vary from patient to patient and across various stages of each person’s treatment. Initial treatment goals might include reduction in acute care utilization (ED visits) and prevention of iatrogenic harm from unnecessary interventions. Goals can then progress to bringing patients into remission. In such a scenario, quality of life improvements and cost savings can be achieved even if patients are not cured, but instead helped to cope or “live with” FND.

Prospective studies that evaluate natural history will assist clinicians in making more accurate and confident diagnoses; for example, exploring the percentage of patients that spontaneously remit, develop new symptoms, or whether symptoms return after remission. Another valuable area to explore would be predictors of FND (biological, social, and cultural factors) by comparing patients with unaffected family members or predictors of recovery. Finally, new tools should be developed to assess outcomes for determination of successful treatment. Tools might include accelerometry, motion-capture devices, and improved measures for quality of life, pain, anxiety, and mood. As discussed below, research evaluating new multidisciplinary models of care and standardized joint-training curriculum should also be conducted in order to generate adaptable care and training models.

### Create Opportunities for Patient Empowerment

The professional’s expertise in managing health-care services does not necessarily translate to insight into the social sphere surrounding the patient’s experience. Patients, families, and advocates must also help drive research priorities. FNDHope, the first non-profit advocacy organization for patients with FND originating in the UK, has already begun to create a database of patients interested in participating in future studies. Such a database not only addresses challenges in small recruitment pools for FND studies but also engages patients as participants as well as advisors in creating a future research agenda.

Part of clinician education must require all stakeholders, especially those patients who live with FND and can share insights into their lived experience. Patient and physician perspectives, collected through qualitative interviews and surveys, can inform new research directions, highlight conceptual struggles, and on-the-ground areas for improvement in clinician training, diagnosis, and treatment (42–47). Education programs that involve direct contact with patients, outside of the clinic, have been shown to reduce stigma perceptions of “otherness” promoting deeper understanding and appreciation for these disorders (48). Such an approach makes the patients part of the conversation, which is empowering. Parity (not pity) and empowerment of individuals who are of the stigmatized group, in this case those with FND, who already feel captive to their bodies, can feel hope and enabled to pursue their life goals in a self-determined way. We have begun implementing this approach to highlight personal narratives at our inaugural conference, where we included the president of FNDHope and also plan to continue and expand upon this tradition by inviting paired clinician–patient narratives in our 2017 conference as well as including patient, family, and advocate representatives.

### Reduce Professional Isolation through Training

Care providers, not unlike their patients with FND, often feel they are in a microcosm of ambiguity and frustration. Clinicians often have few resources having likely received little formal training and even less time to care for these patients and no sense of solidarity and community. So how might we do better to support our clinicians?

While there are some efforts toward clinician community building and training for FND (for example, the Functional Neurological Forum http://fnforum.org), our group felt a conspicuous lack of formalized curriculum in FND in medical school, residency, and beyond. A robust multifactorial approach to FND education can offer providers, patients, and advocates an arsenal of evidence and facts to dispel myths and stereotypes that undermine care for these patients. This would include not only the latest neurophysiological research and exploration of psychotherapeutic approaches but also social science data such as narratives about patient and family experiences and how stigma impacts clinician practices.

Acknowledging to patients that FND is a significant medical condition requiring intervention for improvement of symptoms is of utmost importance. Word choice (including what is said and left unsaid) can have enormous impact on the therapeutic encounter both for better and worse (43). Confronting patients with FND can be accompanied by powerful feelings of vulnerability, helplessness, and frustration. Incorrect physician (and perhaps even patients’) assumptions and biases (i.e., “FND only impacts young women.”) can impact rate of diagnosis and treatment programs (49). There is shame associated with FND both from patients who have internalized the stigma of their diagnosis and from the clinicians (50) who are not quite sure how to care for them once the diagnosis is given (a diagnosis for which they may have their own doubts). The path forward is not simple. Stigma and tolerance of ambiguity must be discussed openly in training and amongst treating clinicians and patients. Clinicians must challenge their own underlying views on functional disorders. They must ask how uncertainty and discomfort with FND patients, or how privileging of the biological over mental disorders, might contribute to their felt or enacted stigma toward FND.

Because the disclosure of diagnosis can be complex, the use of a standardized non-judgmental script, such as outlined by Stone (51) as well as external review of the delivery of that script should be a critical part of ongoing training efforts. A video repository demonstrating strategies of delivering the diagnosis and communication with the patient could be integrated into training. Training modules for medical students and residents—similar to those created by the Accreditation Council for Graduate Medical Education (ACGME) (52)—could be adapted for discussing the complex nature of FND diagnosis and treatment as well as how to respond to challenging questions (“so you are saying this is all in my head,” “you don’t believe me?”).

Joint and cross-disciplinary training about FND for all members in the health-care team should be emphasized and started as early as medical school. Recent cross-disciplinary training efforts within the ACGME Psychiatry and Neurology Milestone Projects (53, 54) are a positive direction for FND training. Medical student/resident training is debated as being quite full (55); however, strategies as simple as providing greater emphasis on existing training such as bedside teaching and modeling would be helpful. Additional modules could also be built into continuing education programs for any health-care provider who might encounter patients with FND including psychologists, social workers, physical therapists, nurses, general practitioners and ED physicians, surgeons, and any physician who might encounter a patient with FND.

### Establish Collaborative Multidisciplinary Care Teams

We propose that a multidisciplinary care team would provide the best opportunity for advanced treatment for patients with FND. While not without its challenges, patients with FND would most benefit from such coordinated care where a consistent message is conveyed avoiding the often present ambiguities currently seen that confuse the patients. In alignment with the most recent DSM-V revision on conversion disorder (56), many conference participants believed that neurology was an important point of entry for patients particularly for their expertise in diagnosing these disorders. However, after this initial referral, the coordination between specialists must be seamless and communication open. Some existing efforts of collaborative teams include a 2012 report from the National Health Service in Scotland that calls for a “integrated neuropsychiatry and clinical neuropsychology service” that includes a team of neurologists, neuroscience nurses, liaison psychiatrists and neuropsychiatrists, clinical neuropsychiatrist, rehabilitation service, and such an collaborative approach continues to be recommend (57, 58). Members of our organizing committee are piloting a multidisciplinary specialty clinic wherein patients, who have received an initial referral from a neurologist, are able to receive a complete evaluation and delivery of diagnosis by a care team including a neurologist, psychiatrist, psychologist, physical therapist, and social worker. The multidisciplinary team creates a treatment plan tailored to fit the unique history and manifestation of the patient’s symptoms. For example, the behavioral health component might include a biopsychosocial assessment as well as recommendations for concomitant mood symptoms by a psychiatrist. Follow-up referrals for mental health treatment could be made in coordination with referrals to other specialties including physical therapy, occupational therapy, and other related fields. Some of the authors have found that follow-up, even as simple as routine phone calls, can help patients become more responsive to recommendations and have significantly better outcomes (59). Just as the neurologist is a critical point of entry into care, the neurologist is also critical for monitoring care. Ultimately, the neurologist must “take ownership” of the patient’s care. The neurologist must be afforded the time to maintain patient contact and monitor the care process in follow-up visits. Incorporating telemedicine for long-term follow-up might be beneficial for patients who do not live in close proximity to specialists on the care team. Annual evaluations in the interdisciplinary clinic might also be considered if needed. The group also proposed a set of simple instructions, such as a flow chart, as a resource for ED doctors, and other first line providers that can avoid fragmented care and harm to these patients. Physicians who frequently care for patients with FND also need their own support by working in a team and perhaps the formation of support groups. Having a trusted team can help decrease care and reduce provider burnout.

A clear hurdle in improving care for patients with FND is the gap in detailed health economics analysis. What are the cost burdens? Who is bearing such costs? While health-care systems should be in the business of promoting wellness, they are equally concerned with cost avoidance. Basic health-care economics research needs to be conducted to help compel structural changes in the current health-care system, which is failing patients with FND. One critical area for future research would include pre- and post-intervention assessments, particularly in the context of a multidisciplinary treatment model. Such assessments could aid in determination of the cost of caring of an individual patient with FND in the 12 months before and after intervention. Similar research in palliative care was able to demonstrate dramatic savings for US hospitals with investments in palliative care (60), and we believe the same can be achieved with a multidisciplinary model for FND. A critical component to this analysis would include assessment of clinician, patient, and family perspectives in order to recognize successes as well as areas for improvement. This data can also facilitate the creation of an exportable model of care for patients with FND.

## Concluding Remarks

We believe it is time to transform the status of FND from a crisis to a priority. A path forward would involve (1) setting a multifactorial research agenda that equally prioritized access to effective psychotherapy as well as identification of novel biomarkers; (2) empowering patients with FND to be heard and to drive changes in care; and (3) reducing isolation for clinicians by providing formal training and setting up multidisciplinary care teams, and support networks. Coordination of care and cross-disciplinary training would greatly facilitate patient wellness by addressing the multifactorial nature of FND and avoid redundancy in gaps of care. All of these advances must be accompanied by frank and honest discussions about stigma rather than a circular conversation about whether FND is a neurological or psychiatric disorder or what name it should bear while solutions are co-created with patients, families, and advocates.

## Author Contributions

KR, SF, SL, PR, RY, and MR contributed to drafting the work or revising it critically for important intellectual content.

## Conflict of Interest Statement

KR, MR, PR, and RY: none. SF: Honoraria: Chelsea Therapeutics, Neurocrine, Lundbeck, Auspex/Teva, Avanir, Cynapsus; Grants: Ipsen, Allergan, Medtronics, Auspex, US World Meds, Pharm-Olam, Cynapsus Therapeutics, Solstice, CHDI Foundation, Michael J. Fox Foundation, NIH; Royalties: Demos, Blackwell Futura for textbooks. SL: Royalties from Demos publishing, editor of “Handbook of ICU EEG Monitoring.”
