# Supplementary material for: Disentangling Stigma from Functional Neurological Disorders: Conference Report and Roadmap for the Future
Source: Front Neurol. 2017 Mar 29;8:106. doi: 10.3389/fneur.2017.00106 (PMC5372777; doi:10.3389/fneur.2017.00106)
Supplement: Supplementary file 1 [file Data_Sheet_1.DOCX]

**Box 1 Patient Narrative**

Jake suddenly developed a constellation of symptoms including stumbling gait, jerking movements, and impaired speech. Alarmed and concerned, his family rushed him to the emergency department (ED). The ED physician told him awkwardly that they could find no abnormalities on his evaluation, that they could not explain his illness, and recommended a neurologist. The neurologist indicated to him that his symptoms were likely psychological and that in fact, this was good news because it was not serious. He was then instructed to see a psychiatrist. Jake reluctantly went to the psychiatrist who said, “You don’t need to see me; you have no clear psychiatric illness yet very clear physical manifestations.” Jake left feeling embarrassed and angry, believing “the docs think this is all in my head and that I’m making this up.” Jake continued to see many providers looking for an answer. They appeared to dread seeing him and wondered if he was malingering or feigning illness. Jake and his family were left feeling frustrated and helpless. His symptoms remained unresolved and he has now amassed tremendous healthcare expenditures.

**Box S1 Breakout Group Prompts and Questions**

**Guiding contexts for discussion**

1. ***Methods of Diagnosis:***

While there is a consistent recommendation against considering FND a diagnosis of exclusion ^1^, objective diagnostic tools remain largely in developmental phases for many functional neurological disorders. The conference participants evaluated current diagnostic approaches for functional movement disorders (FMD) and psychogenic non-epileptic seizures (PNES) as well as gaps in knowledge and methodologies. For example, video-EEG can facilitate PNES diagnosis, whereas movement disorders diagnosis largely relies on observation. Of critical importance is the manner in which diagnostic information is conveyed to the patient. While consistent recommendations in the literature describe using an empathic approach, in reality clinicians often stumble with regard to achieving the appropriate tone ^2^. Further, a distinction should be made between the concept of diagnosis delivery as a treatment strategy versus the diagnosis representing the sole treatment (i.e. delivering the diagnosis will reveal to the patient the true nature of their disorder and they can simply stop their symptoms by realizing they are not “real”). This misconception represents a serious controversy about the nature of the disorder (intentional or not; biological or mental, etc.)

**The Methods of Diagnosis breakout group addressed the following points:**

1. What is the quality of current diagnostic techniques (clinical assessment, EEG etc.) in evaluating PNES and other functional disorders?
2. How can clinical physiology or other laboratory findings be helpful with diagnosis?
3. How can we navigate the diagnostic challenge of differentiating factitious disorders, and malingering from conversion disorder?
4. How can we teach clinicians how to effectively communicate the diagnosis of a functional disorder?
5. ***Methods of Treatment***

While numerous treatment options for FND have been discussed in the literature, placebo-controlled trials are limited to a handful of small cohorts of PNES patients ^3^. No placebo-controlled trials exist for FMD or other FNDs. Current treatment methods and the evidence to support such methods were evaluated with the goal of developing standardized management recommendations. Of critical importance, treatments that have little success, but are used as stop gap measures in the absence of available resources (time/funds/expertise) were discussed. Because patients are particularly suggestible, early interventions can result in better short term outcomes. Therefore, participants also discussed ethical guidelines for the use or abuse of suggestibility.

**The Methods of Treatment breakout group addressed the following points:**

1. How do we tell the patient the diagnosis (the first step to treatment)?
2. What treatment modalities have been utilized successfully and what are the barriers to implementing these treatments?
3. How do we develop multidisciplinary models of therapy?
4. How do we develop clinical trials and outcome measures as the symptoms and causes are heterogeneous?
5. ***Training Physicians/Missing Curriculum****:*

There is a large discrepancy between the prevalent nature of these conditions and the amount of training received in medical school and residency. The need to develop standards of training with consistency between disciplines of psychiatry and neurology was discussed. This included a dialog about terminology as well as exploration of explicit and implicit training processes throughout medical education. The discussion focused on two domains: (1) advancing the medical/technical expertise required to arrive at the appropriate diagnosis of functional disorders and (2) cultivating sensitivity in approaching this patient population. Both domains have implications as to the order of treatment and ultimate responsibility for patients at various stages of care.

**The Curriculum breakout group addressed the following points:**

1. What is the current standard for training neurologists and psychiatrists regarding clinical features, examination, diagnosis and treatment for functional neurological disorders? Is there any standardized curriculum?
2. What are key elements of a proposed curriculum for training neurologists and psychiatrists about functional neurological disorders?
   1. Diagnostic? (including how to deliver the diagnosis)
   2. Treatment? (Who should be trained to treat these patients and how?)
   3. Addressing stigma
3. What other medical and health professionals should receive training and how?
4. What are the most significant “gaps” in how these disorders are currently taught?  How do we start to bridge this gap?
5. ***Healthcare System/Infrastructure Challenges****:*

Participants sought to formulate standards for successfully guiding patients and their healthcare team through the healthcare system that is still learning how, or perhaps simply is currently unfit, to accommodate an abundance of patients with medically unexplained illnesses. Participants discussed systemic hurdles within their own practices and discussed possible actionable solutions for change. This discussion included how to best utilize a multidisciplinary approach to treatment and hurdles related to the feasibility of such a method. This would also be divided into two domains: diagnosis and treatment. For the diagnostic domain, the focus was on identification of patients (including acknowledgement of the patient/family) and referral to the appropriate team for diagnosis (i.e. mental health practitioners, internal medicine, neurology and even more specifically movement d/o vs. epilepsy specialist). For the treatment domain, the focus was on treatment options, which may move beyond neurology’s area of expertise. For example, physical therapy and behavioral interventions may be extremely costly and lack individuals or difficulty in identifying individuals who might have the appropriate expertise. In such cases how might the neurologist and/or internist feasibly play a role in the treatment process?

**The Infrastructure Challenges breakout group addressed the following points:**

1. How do patients with functional disorders impact health systems and how do these disorders affect cost structures?
2. How do systems handle such patients today?
3. Are there better approaches we should be taking?
4. What are the obstacles to putting in place these best practices or different approaches?

**References**

1. Gupta A, Lang AE. Psychogenic movement disorders. *Curr Opin Neurol.* 2009;22(4):430-436.

2. Monzoni CM, Duncan R, Grunewald R, Reuber M. How do neurologists discuss functional symptoms with their patients: a conversation analytic study. *Journal of psychosomatic research.* 2011;71(6):377-383.

3. LaFrance WC, Jr., Keitner GI, Papandonatos GD, et al. Pilot pharmacologic randomized controlled trial for psychogenic nonepileptic seizures. *Neurology.* 2010;75(13):1166-1173.
